# The role of complement in long COVID pathogenesis

**DOI:** 10.1172/jci.insight.194314

**Published:** 2025-08-22

**Authors:** Rafael Bayarri-Olmos, William Bain, Akiko Iwasaki

**Affiliations:** 1 Howard Hughes Medical Institute, Chevy Chase, Maryland, USA.; 2 Department of Immunobiology and; 3 Center for Infection and Immunity, Yale University School of Medicine, New Haven, Connecticut, USA.; 4 Division of Pulmonary, Allergy, Critical Care, and Sleep Medicine, University of Pittsburgh School of Medicine, Pittsburgh, Pennsylvania, USA.; 5 Veterans Affairs Pittsburgh Healthcare System, Pittsburgh, Pennsylvania, USA.

## Abstract

Long COVID is a debilitating condition that can develop after a SARS-CoV-2 infection and is characterized by a wide range of chronic symptoms, including weakness, neurocognitive impairment, malaise, fatigue, and many others, that affect multiple organ systems. At least 10% of individuals with a previous infection may develop long COVID, which affects their ability to perform daily functions and work. Despite its severity and widespread impact, this multisystemic condition remains poorly understood. Recent studies suggest that dysregulation of the complement system, a key component of the innate immune response, may contribute to the pathogenesis of long COVID, particularly in connection with coagulation, inflammation, and vascular injury. In this Review, we examine the evidence linking complement system dysregulation to long COVID and explore its potential role in driving disease pathology.

## Introduction

Four hundred million people worldwide are believed to have developed long-term health issues after COVID (1). These individuals suffer from an often debilitating condition that has become known as long COVID, which is estimated to affect around 10% of people with a previous SARS-CoV-2 infection. Patients with long COVID report a wide range of symptoms, such as weakness, malaise, fatigue, and brain fog, spanning almost every organ system with significant impacts on quality of life (2–5). Many find their ability to perform daily activities severely impaired and require accommodations to return to work, while a significant proportion of people with long COVID are not able to work at all (4). Long COVID is also associated with an increased risk of new-onset conditions, such as cardiovascular disease and diabetes (6, 7). The broad symptomatology reported by patients with long COVID overlaps with other poorly characterized long-lasting illnesses, like myalgic encephalomyelitis/chronic fatigue syndrome, and other post-acute infection syndromes, including long SARS (8, 9). These syndromic overlaps contribute to ongoing challenges in defining the prevalence and etiology of long COVID.

Despite the dramatic impact of long COVID, there are no proven diagnostic or therapeutic tools available. To this end, investigation of long COVID pathogenesis is a high priority. Several hypotheses regarding the root causes that drive long COVID have been proposed, including abnormal immune responses during acute SARS-CoV-2 infection, a persistent viral reservoir (or persistence of viral antigens or RNA), reactivation of latent viruses, microbial dysbiosis, unrepaired tissue damage, and autoimmunity (10–14). Notably, the proposed root causes of long COVID are not mutually exclusive, and multiple etiologies may contribute to the condition in some patients, which may account for the diverse symptomatology and clinical presentation. Importantly, the complement system, which is a crucial component of the immune response to viral infection and contributes to immune roles in tissue repair, has potential mechanistic roles in each of the proposed root causes of long COVID. Indeed, recent studies have suggested hyperactivation of the complement system as a pathological mechanism in long COVID (15–20). Here, we examine the evidence linking dysregulation of the complement system and its intimate relationship with vascular injury to the pathogenesis of long COVID.

## The complement system

The complement system is one of the first and evolutionarily oldest lines of host defense. Dating back more than 600 million years — well before the emergence of vertebrates and the adaptive immune system — this ancient mechanism has coevolved to form a finely tuned enzymatic cascade to combat microbial pathogens (21). The complement cascade comprises more than 50 soluble and membrane-bound proteins, contributing not only to the clearance of pathogens but also to various other biological processes, such as coagulation (22). There are three distinct, yet intertwined, pathways of complement activation: the classical, lectin, and alternative pathways. All three pathways lead to the cleavage of the most abundant complement protein and the main complement effector, C3 (23). However, the activation steps leading to C3 cleavage differ depending on the pathway trigger.

**Figure 1:**
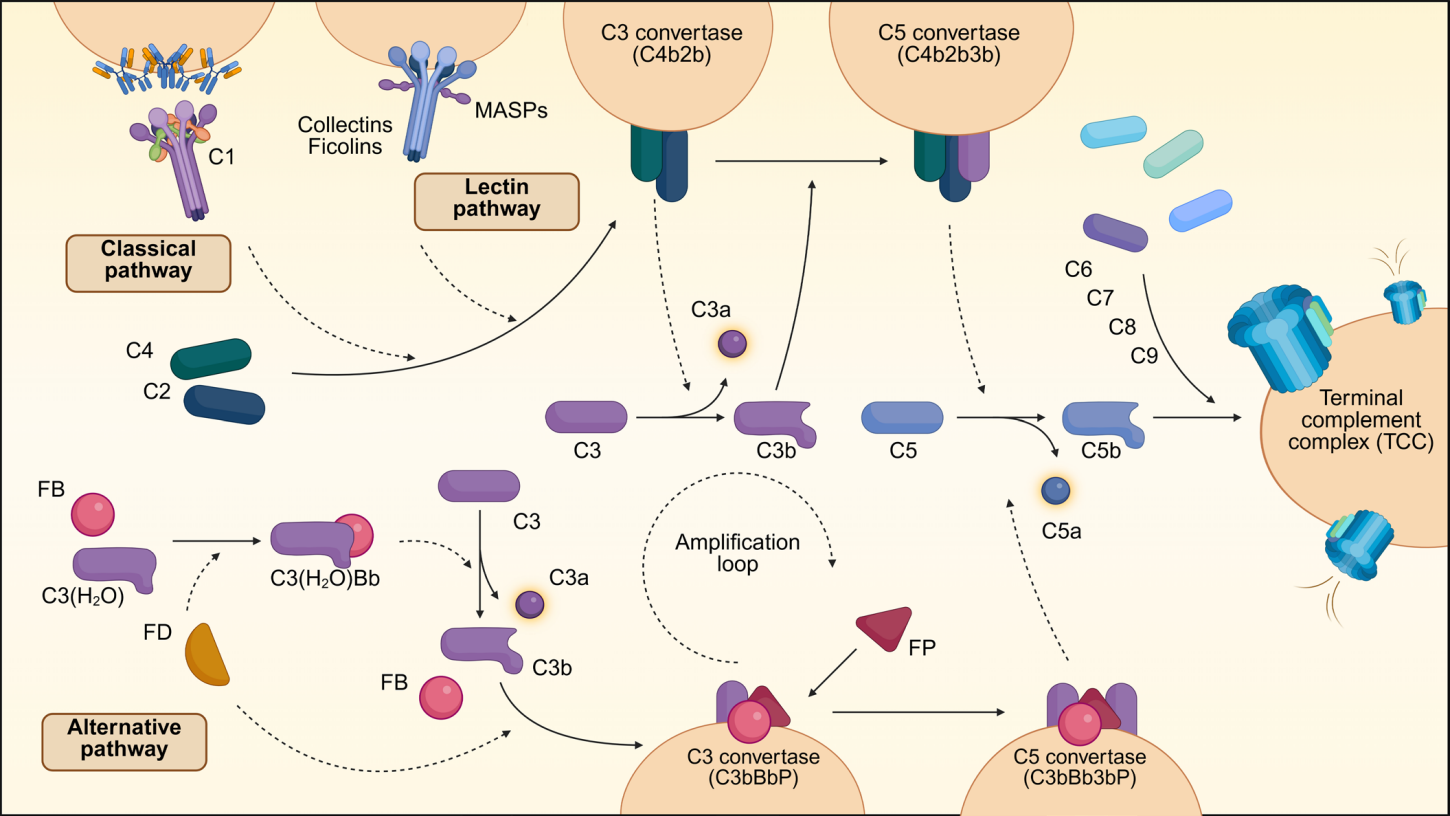
Overview of the complement system. The complement cascade can be initiated by three distinct pathways: the classical, lectin, and alternative pathways. The classical and lectin pathways are triggered when recognition molecules bind to structures such as antibody complexes and carbohydrates on pathogen surfaces, leading to the activation of their associated proteases C1s/C1r and MASP-1/2. These proteases cleave C4 and C2, generating the C3 convertase (C4b2b), which then processes C3 into the anaphylatoxin C3a and the opsonin C3b. Accumulation of C3b induces the formation of the C5 convertase, which cleaves C5 into C5a and C5b. The subsequent interaction of C5b with C6, C7, C8, and C9 leads to the assembly of a lytic pore, known as the terminal complement complex (TCC). The alternative pathway is initiated when factor B (FB) interacts either with water-hydrolyzed C3, C3(H_2_O), or with deposited C3b to form the C3(H_2_O)Bb or C3bBb C3 convertases following factor D (FD) cleavage. These proteases, and in particular, the surface-bound and properdin/factor P–stabilized (FP-stabilized) C3bBbP C3 convertase act as an amplification loop for complement, generating most of the activated C3 fragments regardless of the initiating pathway.

The classical pathway of complement activation is initiated by binding of the pattern recognition molecule C1q to pathogen surfaces or to the Fc region of antigen-bound IgG or IgM. C1q, together with the serine proteases C1r and C1s (C1r_2_C1s_2_), comprise the C1 complex, which circulates in the bloodstream to perform surveillance. Upon binding, the C1 complex undergoes a conformational change that results in the autoactivation of C1r, which in turn cleaves and activates C1s (24). Activated C1s cleaves C4 and C2, releasing the C4a and C2a fragments into the circulation and generating a C3 convertase, C4b2b (formerly known as C4b2a) (25), which remains bound to the pathogen surface (Figure 1).

The lectin pathway of complement activation yields the same C4b2b convertase as the classical pathway but is initiated by the binding of the pattern recognition molecules, such as collectins and ficolins, to carbohydrates that are enriched on the surface of pathogens or apoptotic cells (26). Collectins and ficolins form bouquet-like structures, much like C1q, with globular heads that bind to their respective ligands and collagen stalks that interact with the serine proteases MASP-1 and MASP-2. These serine proteases are homologous to the C1r/C1s proteases of the classical pathway (27). Collectins and ficolins deposit on the target surface, leading to the autoactivation of MASP-1, which subsequently activates MASP-2 (28). Both activated MASPs cleave C2, while MASP-2 cleaves C4. These cleavage events generate membrane-bound C3 convertase C4bC2b (Figure 1).

In contrast with the classical and lectin pathways, the alternative pathway is unique because it is constitutively active and able to amplify the other two pathways (29, 30). The alternative pathway can be initiated on cell surfaces when C3b binds factor B to form the C3bB complex. C3b-bound factor B can then be cleaved by factor D, releasing the small fragment Ba and forming the alternative pathway C3 convertase C3bBb. The alternative pathway is also constitutively and spontaneously activated in the circulation by a separate trigger known as “C3 tick-over” (31). To protect against self-attack, host cell surfaces are decorated with complement regulators that can rapidly degrade C3b into iC3b, which is incapable of propagating the complement cascade (20). In contrast, foreign surfaces, such as bacteria, lack complement regulators, and thus C3b deposition results in full-fledged alternative pathway activation, tagging foreign surfaces for elimination by effector cells (Figure 1).

The three complement activation cascades converge on the generation of C3 convertases that cleave C3 to generate C3b and C3a. C3b exposes a transient thioester that can form a covalent amide and ester with proteins and carbohydrates. The half-life of this highly reactive intermediate is very short (around 100 μs), and thus C3b deposition is restricted to the vicinity of the convertase. Clustering of newly formed C3b molecules causes the specificity of the convertase to shift dramatically toward C5, which forms the C5 convertase, the first step of the terminal pathway (32, 33). Activation of C3 and C5 by their respective convertases releases C3a and C5a into the circulation. These small fragments, known as anaphylatoxins, mediate chemotaxis and activation of immune and nonimmune cells (34). Cleavage or conformational activation of C5 allows the binding of C6, which then can associate reversibly with the cell membrane of the target cell to form the C5b6 complex (35). This complex further recruits C7, C8, and several copies of C9 to form the terminal complement complex (TCC) or membrane attack complex, a transmembrane pore that causes an influx of water and subsequent cell lysis and death (35) (Figure 1). Notably, recent research has identified an additional pathway of cell surface complement activation via lymphocyte-derived granzyme K that deserves additional study but will not be addressed in this Review (36).

## The complement cascade, coagulation, and vascular injury

In addition to its role in the response to pathogens, complement is increasingly recognized for its intimate relationship to the coagulation cascade. In fact, complement and coagulation are host defense networks with a common evolutionary ancestor (37). These shared origins are reflected in their many similarities and extensive cross-communication. Both are blood-based protein cascades that can be proteolytically activated in response to different triggers, such as infection, working in concert to restrict the spread of pathogens and recruit and activate effector cells.

One example of complement-coagulation crosstalk occurs through ADAMTS13 (ADAM metallopeptidase with thrombospondin type 1 motif 13) and vWF, which are both involved in platelet and thrombus formation and can directly regulate complement at the endothelial surface. Endothelial cells and megakaryocytes store vWF for release into the bloodstream upon contact with activating stimuli (38). ADAMTS13 cleaves vWF multimers into smaller forms that act as cofactors for the complement regulator factor I (FI) in the inactivation of C3b into iC3b (39). Once the complement cascade progresses to the TCC stage, C5b–9 insertion on the membrane results in further secretion of high–molecular weight vWF multimers (40). Importantly, the anaphylatoxins C3a and C5a generated during complement activation can interact with their cognate receptors on endothelial cells and immune cells to promote the release of various pro-inflammatory and procoagulant molecules, including IL-6 and tissue factor (41–45), which may amplify pathology and vascular injury. Elevated levels of IL-6 and thrombospondin 1 (TSP1) may contribute to the formation of complement-activating large vWF multimers by disrupting ADAMTS13-vWF interactions (46–48). Finally, vWF and other molecules released by activated endothelia can recruit, activate, and anchor platelets that may contribute to thrombosis and additional complement activation.

Another example of complement-coagulation crosstalk involves stimulated platelets that can tether C3(H_2_O) via P selectin or properdin (49, 50). C3(H_2_O) bound to the platelet surface serves as a ligand for the CD11b/CD18 complex (also known as complement receptor 3) and can mediate monocyte-platelet aggregate formation (51). Platelets can also be activated by C1q via the C1q receptor expressed on the platelet surface (52). This interaction results in the expression of P selectin and induction of platelet procoagulant activity. If the complement cascade continues, as seen in patients with defects in complement regulators or cases of exaggerated complement activation, such as sepsis, C5b–9 inserts on the platelet membrane and triggers the shedding of membrane vesicles (53, 54). These vesicles express binding sites for the FXa/FVa pro-thrombinase complex and thus can induce platelet procoagulant activity. Moreover, complement can be directly activated by various proteases within the coagulation cascade, bypassing the canonical activation by pattern recognition molecules of the classical and lectin pathways (37).

This intimate relationship between complement and coagulation, when dysregulated, can lead to vascular injury, including thrombotic microangiopathies, such as atypical hemolytic uremic syndrome and systemic lupus erythematosus (55, 56). Due to its role in antiviral responses, complement activation is now also known to be a hallmark of host response to SARS-CoV-2 infection (57, 58). However, the complement antiviral response may become exaggerated and maladaptive, which along with a hypercoagulable state, has been associated with disease severity and adverse outcomes in COVID-19 (19, 58–65). As with many aspects of the host immune response, too much of a good thing can have harmful consequences; therefore, we next consider the potential role of complement dysregulation in long COVID.

## Considerations for complement testing

Given the prominent role of complement in inflammatory diseases and immunodeficiency disorders, complement testing is routinely performed in the clinic and many research laboratories (66–68). However, the autoproteolytic cascade of complement and lack of a readily available calibration standard are challenges for reliable implementation and interpretation of complement assays.

Functional assays are often the first line of testing when complement deficiencies and other disorders involving complement are suspected (Table 1) (69). These include classical (CH50), which is FDA approved, and alternative (AH50) pathway hemolytic assays; liposome-based tests that circumvent the need for fresh erythrocytes; and pathway-specific functional ELISAs suitable for high-throughput screening of complement functional deficiencies (69, 70). These assays are best performed using serum, as anticoagulants like EDTA, and to a lesser extent citrate, chelate Ca^2+^ and Mg^2+^ cations required for activation of complement, and heparin may interfere with the function of several complement components (71).

Quantitative assays of plasma can determine the concentration of the relevant complement component(s). The most common assays are immunoprecipitation-based assays, such as nephelometry and turbidimetry, and ELISA (67). However, assays using mAbs that target epitopes exposed only after activation of zymogen molecules (neoepitopes) are required to assess activation. For example, the mAb aE11, which detects a neoepitope in activated C9 (72), has been used extensively for the past 40 years to detect complement activation in plasma (70, 73). Quantification of complement components and activation fragments is best carried out using plasma, preferably EDTA- or hirudin/lepirudin-treated plasma, to minimize coagulation-mediated complement activation (67, 74).

Other important considerations when designing and analyzing complement assays are the half-lives and handling tolerance of the selected analytes. For example, C3dg, which has a longer half-life than C3a, is often preferred when evaluating C3 activation (75).

Regardless of assay type, serum and plasma should be processed promptly, avoiding prolonged exposure to room temperature, which results in ex vivo activation of the complement cascade (76, 77). Processed samples should be stored at −80°C, with limited freeze-thaw cycles. For a more in-depth discussion on this topic, the reader is directed to several excellent reviews (66, 67, 69, 78). Often, best practices for complement analyses conflict with the practical limitations of biobanking. In such cases, results must be interpreted with caution, acknowledging the potential for preanalytical artifacts, such as ex vivo activation.

## Complement signatures in long COVID

There is a growing body of work that shows an association between immune system dysregulation, persistent inflammation, and long COVID (79–84). Although the focus of these studies has largely been on myeloid and lymphoid cell populations and associated cytokines, chemokines, and receptors, the complement system is intricately involved in many of the steps of the inflammatory response (85). For the purposes of this Review, we focus on available reports that directly study the association between complement and the post-acute phase of COVID-19 (Table 2). We highlight seven studies addressing complement activation, complement function, complement biomarkers, and viral biomarker signatures.

### Complement activation.

In a comprehensive multicenter longitudinal study, Cervia-Hasler and colleagues followed a cohort of 113 patients who had COVID-19 and 39 healthy controls for up to a year to identify biomarkers associated with the development of long COVID (15). At 6-month follow-up, 40 patients exhibited long COVID, which was defined as 1 or more persisting COVID-19–related symptoms. Using the SomaScan platform — a high-throughput proteomics technology that employs synthetic aptamers to target over 6,500 unique proteins — and mass spectrometry, the authors identified persistent complement activation in sera from patients who developed long COVID. The authors found elevated levels of alternative pathway activation markers Ba, a split product of factor B cleavage, as well as C3d, which can be released from fluid-phase cleavage of C3 products during activation. Additionally, the authors observed a reduction in C7-containing TCCs in patients with long COVID and concluded that this reduction indicated increased formation of membrane-bound TCCs. Notably, the distinction between C5b6- and C7-containing complexes was made possible by the identification of an aptamer that preferentially binds to complexed C7, rather than monomeric C7. The capacity to differentiate intermediate forms of the TCC provides insights into complement activation dynamics on surfaces and in the fluid phase. However, this assay requires further independent validation. It is also important to note that plasma is preferred for measurement of complement activation rather than serum specimens, as utilized in this study, which retain the capacity for ex vivo proteolytic activation (76, 86).

**Figure 2:**
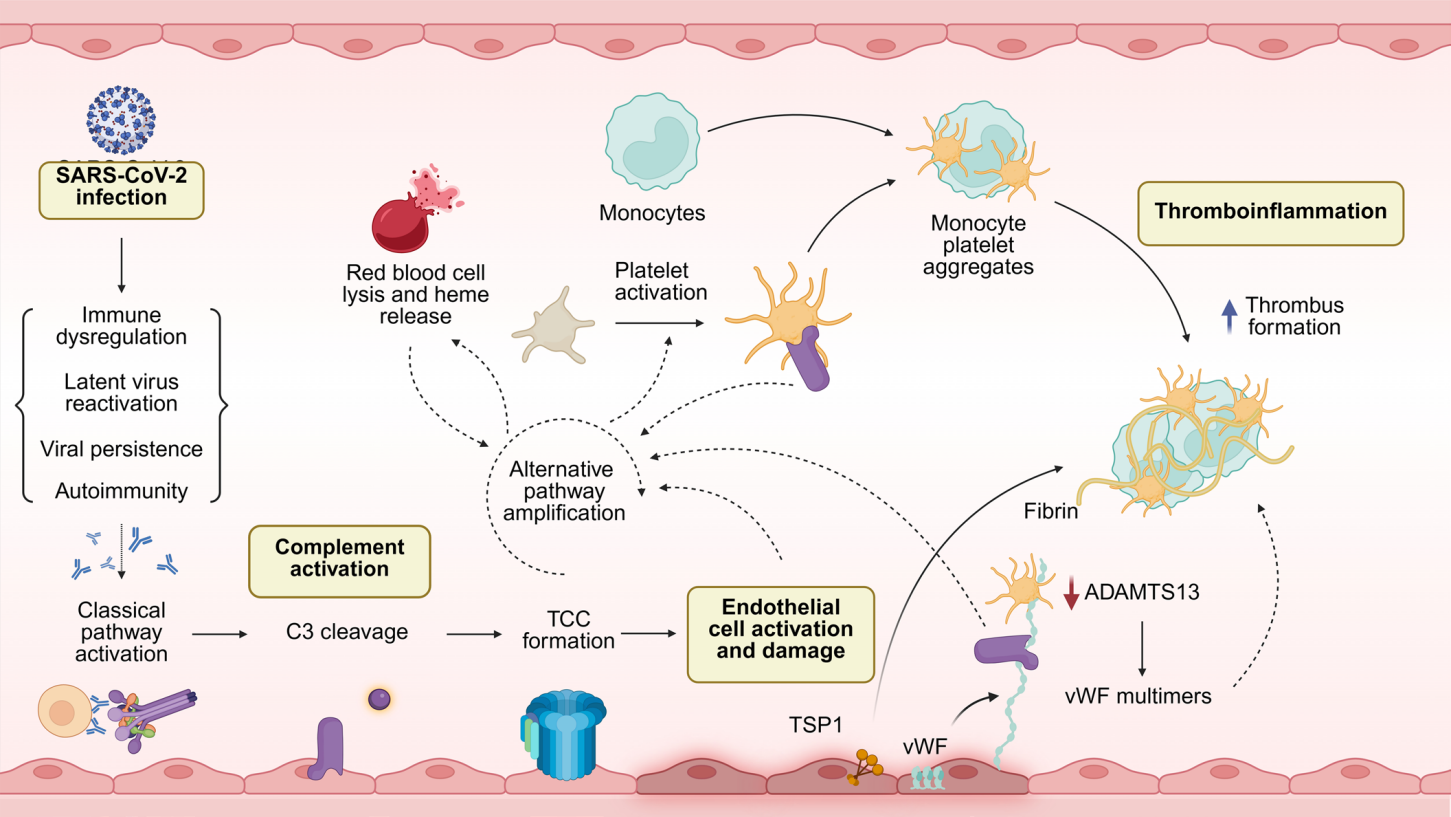
Proposed drivers of complement activation in long COVID. SARS-CoV-2 infection triggers activation of the complement cascade through direct interaction with viral components or virus-specific antibodies, typically resolving once the infection is cleared. However, in patients with long COVID, this activation may persist, potentially contributing to ongoing symptoms. Several proposed mechanisms of long COVID can directly activate the complement system. For example, antiherpesvirus antibodies, likely the result of herpesvirus reactivation, or autoantibodies may drive activation via the classical pathway. Insertion of TCC in the endothelial cell wall causes activation and cell damage, causing the release of TSP1 and vWF. TSP1 promotes formation of monocyte-platelet aggregates, while vWF release — coupled with reduced levels of ADAMTS13, the metalloproteinase responsible for processing vWF multimers — leads to the accumulation of large or ultralarge vWF multimers on the endothelial surface. This, in turn, promotes platelet recruitment and thrombus formation. Additionally, vWF multimers on the endothelium, along with properdin and P selectin on activated platelets, can trap C3b, fueling complement activation via the amplification loop of the alternative pathway. Uncontrolled complement activation in the vasculature leads to red blood cell lysis, causing the release of heme and activation of the alternative pathway. Finally, tissue damage resulting from acute COVID-19, autoimmunity, or viral antigen reservoirs may all contribute to the persistent complement activation observed in patients with long COVID.

In a cross-sectional study published shortly after Cervia-Hasler et al., Baillie and colleagues at Cardiff University analyzed plasma from 166 patients with long COVID and 79 healthy convalescent controls with a history of COVID-19 (16). Using ELISA and machine learning, they evaluated 21 complement biomarkers to identify predictive signatures of long COVID. Participants were well matched across key variables except BMI, which was significantly higher in patients with long COVID. Approximately 50% of participant samples were collected more than 2 years postinfection. Baillie et al. found significantly elevated plasma levels of C1s–C1-INH complex and of Ba and iC3b, markers of activation of the classical and alternative pathways, respectively, along with C5a and TCC, indicative of terminal pathway activation. Generalized linear models were used to identify predictive biomarker sets, and the tractable combination of activation markers Ba, iC3b, C5a, and TCC was found to have strong predictive power for long COVID. These findings highlight activation of the classic and alternative pathways as potential contributors to long COVID, as well as the potential utility of complement activation as a biomarker for diagnosis (Figure 2).

A recent longitudinal study of complement activation profiles during and after the acute phase of COVID-19 by Barratt-Due and colleagues (19) further underscores findings of sustained complement activation. EDTA-treated plasma samples were collected during hospitalization, and at 3 months and 1 year after hospitalization, from 457 hospitalized patients with COVID-19. The authors measured various complement activation products (C4d, C3bBbP, C3bc, C5a, and TCC) by ELISA. Hospitalized patients demonstrated increased levels of complement activation markers, similar to prior reports (63). Even after hospital discharge, complement activation persisted. For example, elevated C4d levels were associated with reversible chest computed tomography changes at 3 months, which the authors suggest may reflect persistent pulmonary inflammation. None of the complement activation products were associated with irreversible computed tomography changes or impaired lung function. Of note, all activation products remained elevated for up to 1 year (*n* = 41).

Another study suggests classical/lectin pathway activation in patients with long COVID (18). In a cross-sectional study that included 152 patients with long COVID from two study sites (mean time from infection 411 days), 37 controls infected before vaccination, 39 controls infected after vaccination, and 40 noninfected vaccinee controls, Klein et al. performed multidimensional immune phenotyping followed by unbiased machine learning analyses to identify factors associated with long COVID. Parallel multiplex analyses of soluble immune mediators in serum identified increased levels of C4b, a key protein in both the classical and lectin pathways that is a potential biomarker of activation, as a distinguishing feature of long COVID. The previous caveat regarding the use of serum for complement activation markers remains relevant here.

In contrast with the studies cited above, which together paint a picture of persistent complement activation during long COVID, a recent preprint suggests that age- and sex-related differences in complement biology may affect these analyses (87). Farztdinov et al. reanalyzed mass spectrometry data published by Cervia-Hasler et al. after adjustment for age and BMI using a balanced factorial design strategy. In this much smaller cohort (29 patients with long COVID and 56 healthy controls), none of the complement components were found to be significantly altered in patients with long COVID. Farztdinov et al. raised concerns that demographic imbalances in age and BMI in the original cohort could be responsible for the reported complement activation signature. In addition, they conducted proteomic analyses from two independent cohorts — one with severe acute COVID-19 (WHO severity grade 3–7) and another with mild acute COVID-19 with persisting fatigue. In the severe acute COVID-19 cohort, there was no evidence of persistent complement activation 6 months after hospital discharge, other than a slight increase in properdin levels in patients with long COVID. In the second cohort, FI was the only complement protein elevated 5–9 months after a mild COVID-19 infection. Instead of persistent complement activation, the authors concluded that the observed proteomic signatures are indicative of ongoing infection or sustained immune activation.

These discrepancies between research groups are not unexpected (Table 2). Variability in study design, methodology, and patient demographics likely contribute to these differences. For example, proteomic analyses of plasma complement signatures may lack the resolution to identify changes in the levels of activated complement fragments. Immunoassays using antibodies targeting neoepitopes present in the active molecules may be a more suitable approach (75).

### Complement function.

Two of the aforementioned studies that highlighted potential complement activation during long COVID also measured complement function. Cervia-Hasler et al. used serum samples and a commercial ELISA and demonstrated increased classical pathway function (15). Interestingly, levels of soluble C5b–9, a commonly used marker of terminal complement activation that requires the formation of both convertases and completion of the complement cascade, remained unchanged. In contrast, Baillie et al. found no difference between patients and controls in a hemolytic assay for classical pathway function (CH50) (16). An important caveat is the use of EDTA-treated plasma specimens in this study. Because EDTA quenches complement proteolytic capacity, it limits the use of plasma for functional assays of complement. In addition to these studies, a retrospective observational study conducted at the Okayama University Hospital (Japan) explored the relationship between complement functional activity and clinical characteristics of patients with long COVID (17). The study by Hagiya et al. included 478 individuals who visited the COVID-19 aftercare clinic, all of whom experienced symptoms lasting more than 1 month after COVID-19 onset. Blood samples were collected approximately 3 months postinfection, and complement activity (CH50) was measured in serum using a commercial automated liposome assay. Based on CH50 level, patients were categorized as having normal or high complement function (CH50-normal vs. CH50-high). Multivariate analyses revealed a significant association between high CH50 levels and brain fog. However, caution should be exercised in interpreting these results, as the absence of a healthy convalescent control group complicates the ability to draw definitive conclusions. Moreover, patients in the CH50-high group were older on average. Considering that complement functional activity positively correlates with age (88), adjustment of the CH50 values for age-related effects should be considered before drawing further conclusions.

### Complement biomarker signatures.

Beyond complement activation markers and functional assays, several groups have assessed additional complement biomarkers for long COVID. A collaboration between Semmelweis University (Hungary) and Hycult Biotech (The Netherlands) set out to investigate whether such biomarkers could be used to predict development of long COVID at the time of initial COVID-19 diagnosis (89). The cohort included 47 healthy controls, 103 patients with moderate COVID-19, and 112 patients with severe COVID-19. EDTA-treated plasma samples were collected soon after infection or symptom onset (9 [5–18] days). Long COVID, defined as persistent symptoms 6–12 months after infection, was observed in 32 out of the 215 patients with COVID-19. The authors investigated the predictive value of complement and macrophage activation markers from the acute-infection phase in the development of long COVID. Plasma levels of complement markers (PTX3, C1q, and C1-INH) and macrophage activation (soluble mannose receptor) were elevated in patients with COVID-19 compared with healthy controls. There was no difference between patients who recovered (healthy convalescent) and those whose symptoms persisted after 6–12 months. As complement activation is a normal physiological response against viral infections, identification of a predictive long COVID signature of complement activation during the acute phase of COVID-19 may prove challenging.

Looking at samples from patients with long COVID 1 to 2 years postinfection, Baillie et al. observed a significant reduction in C1q and a significant increase in C3, C5, and C9 in these patients compared with healthy controls (16). The authors suggested the reduction in C1q is caused by consumption after activation of the classical pathway. They also observed a significant elevation of the plasma levels of several fluid-phase complement regulators, such as C1-INH, an inhibitor of the serine proteases of the classical and lectin pathways, and proteases of the contact system of coagulation (90); factor D, factor H, and properdin, regulators of the alternative pathway; and clusterin, a regulator of the terminal pathway. The upstream signaling that contributes to these broad increases remains uncertain.

In partial disagreement with Baillie et al., a prospective multicenter study by Liew et al. found elevated levels of COLEC12 and C1qA in a subset of patients with long COVID (20). The authors profiled 368 plasma proteins using Olink in 424 patients with long COVID and 233 recovered individuals 6 months posthospitalization. COLEC12 was associated with fatigue, anxiety/depression, and cardiorespiratory and cognitive symptoms. C1qA, which along with B and C chains, forms the multimeric C1q molecule (91), was associated with gastrointestinal and cognitive symptoms. In addition to initiating the classical pathway, C1q modulates endothelial cell activation, immune cell differentiation, and autoimmune and antiviral responses in a complement-independent fashion (92–94). Inflammatory stimuli are known to increase expression of C1q in macrophages (95), and the authors also observed elevated markers of myeloid inflammation (20). Aberrant complement and macrophage activation are hallmarks of pathological responses in COVID-19 (96–98), which may remain unresolved in long COVID.

Finally, Cervia-Hasler et al. demonstrated increased levels of major complement effectors C3 and C5 in patients with long COVID at 6-month follow-up (15). Markers of tissue injury and thromboinflammation were also increased, including elevated levels of heme, vWF, ADAMTS13, and platelet activation markers. Flow cytometry analyses revealed increased monocyte-platelet aggregates in patients with long COVID. Taken together, a compelling interpretation of these data would be that increased complement deposition at endothelial cell membranes leads to cellular and platelet activation, vWF release, and vascular injury that may further fuel a self-amplifying cycle of tissue damage and complement activation (Figure 2). Vascular changes and coagulation abnormalities are linked to poor COVID-19 prognosis (99) and may persist in some patients with long COVID (100). Increased levels of complement components and vWF have been found in microclots derived from plasma of patients with long COVID (100).

### Signatures of viral reactivation.

Although the mechanism(s) for complement activation during long COVID remains elusive, reactivation of latent herpesvirus may contribute. To that end, Cervia-Hasler et al. performed high-throughput antiviral antibody profiling by phage immunoprecipitation sequencing technology (VirScan) (15). They observed increased IgG titers against epitopes from the common herpesviruses cytomegalovirus and Epstein-Barr virus (EBV). EBV reactivation has been implicated in the development of long COVID by other groups (101, 102). The authors proposed that antibodies against herpesvirus trigger classical pathway activation, thus contributing to pervasive immune and inflammatory responses. Consistent with this mechanism, Klein et al. also reported higher antibody responses against herpesviruses, particularly EBV, as measured by ELISA and two high-throughput antibody discovery platforms (serum epitope repertoire analysis and rapid extracellular antigen profiling) (18). However, this study did not directly examine correlation between EBV-reactive antibodies and complement activation. Yet, taken together, these two studies identify viral reactivation as an appealing mechanism for further study.

### Limitations.

A key limitation of these studies is the lack of data on individuals’ pre–COVID-19 complement profiles. Levels of complement components and endothelial activation markers have been shown to be predictive of COVID-19 severity or outcome (59, 63, 103–107). Given that disease severity appears to be associated with increased risk of developing long COVID (108–110), investigating pre–COVID-19 complement levels may help clarify whether complement dysregulation directly contributes to disease susceptibility and long-term sequelae or arises secondarily as part of the disease process. A promising strategy to answer this question is through genetic analyses of the complotype (the set of inherited complement gene variants that influence susceptibility to disease) (111, 112). Polymorphisms and rare mutations are known to affect both the expression and activity of complement components (113), and several studies have already explored associations between complement gene variants and susceptibility to acute COVID-19 (62, 114–121). However, similar studies for long COVID are limited (118, 119). A recent GWAS by the Long COVID Host Genetics Initiative did not identify complement variants reaching genome-wide significance (109). Nonetheless, the dataset, comprising over 15,000 long COVID cases and nearly 2 million controls, remains a valuable resource for follow-up investigations. Complement variants that do not reach genome-wide significance individually may still contribute to susceptibility through polygenic or pathway-level mechanisms.

We also note that measurements of circulating complement components and activated fragments in blood provide only indirect evidence of cellular and tissue activation. For example, T cell–mediated activation of complement is a potentially important mechanism in tissues that may not be captured by systemic or blood-based assays (36). Direct in situ detection of complement fragments and complexes (e.g., iC3b, C5b–9) in the vasculature and other relevant tissues in patients with long COVID would offer more definitive insights. To our knowledge, such data are currently lacking.

Animal models of long COVID have not examined the role of complement. However, mouse models of acute coronavirus infection demonstrate that complement activation contributes to disease severity and lung pathology (122, 123). By analogy, complement-driven inflammation, endothelial and immune cell activation, and tissue damage may play a role in the chronic phase. A sustained activation of the complement-coagulation axis driven by a range of potential stimuli, such as tissue damage from the acute phase, or repeated encounters with persistent SARS-CoV-2 antigens or reactivated herpesviruses, may underlie certain manifestations of long COVID.

Finally, demographic factors, such as age, sex, and BMI, are known to influence the circulating levels of several complement components (88, 124–126), which may confound analysis, as all three factors have been associated with an increased risk of long COVID (3, 110). Adding to this complexity, the heterogeneity of long COVID itself further complicates comparisons. Despite these limitations, the promising data linking complement biology with long COVID suggest that we should consider its clinical implications.

### Clinical considerations: is complement a therapeutic target in long COVID?

We have cataloged existing evidence of complement activation in patients with long COVID, yet it remains uncertain whether this process can be harnessed for diagnostic or therapeutic benefit. Two fundamental questions could be pursued to understand whether complement activation could be targeted during long COVID. First, is complement activation increased during long COVID, and if so, why? Second, can the mechanism of complement activation be therapeutically targeted? We propose two primary hypotheses for the relationship between complement activation and long COVID: 1. Complement activation is a physiological response to a pathogenic stimulus (e.g., viral persistence or reactivation of latent herpesviruses); or 2. aberrant complement regulation yields overexuberant complement activation that is maladaptive during long COVID. These hypotheses are not mutually exclusive but would be helpful to dissect the pathogenesis of long COVID and to effectively deploy precision medicine, including complement therapeutics (127), to those living with long COVID.

There is evidence to support a persistent pathogenic stimulus during long COVID. As described above, others have reported herpesvirus reactivation in long COVID. Furthermore, SARS-CoV-2 is known to activate complement pathways (63, 128), and persistent SARS-CoV-2 infection may drive pathology in a subset of patients with long COVID (10–14). For example, 43% of patients with long COVID symptoms had detectable SARS-CoV-2 spike or nucleocapsid antigen in blood up to 14 months after infection, which associated with long COVID symptoms (129). Similarly, long COVID symptoms have also been associated with persistent SARS-CoV-2 viral RNA in tissue specimens from numerous organ systems (130). Therefore, further definition of potential pathogenic stimuli may be beneficial to understanding whether therapeutics targeting the pathogen (e.g., antivirals) or the host immune response (e.g., complement inhibition) would be most beneficial overall and in individual patients. In contrast, should maladaptive complement activation contribute to long COVID, the potential therapeutic approaches are more promising.

Currently available complement therapeutics generally either directly target complement-mediated inflammation (e.g., anaphylatoxins) or work as inhibitors to restrain overexuberant complement activation. One strong example of the potential for complement therapeutics during COVID-19 infection is the use of anti-anaphylatoxin therapy with an mAb against C5a, which demonstrated a benefit against lethal COVID-19 in the PANAMO trial (131). However, whether anti-anaphylatoxin therapy would be beneficial in long COVID is unclear. Complement inhibitors have shown benefit in several disease states marked by complement dysregulation. For example, C5-targeting mAb eculizumab dramatically improved the care of patients with the complement-mediated thrombotic microangiopathy atypical hemolytic uremic syndrome (132). Other C5 mAbs, such as crovalimab, have shown promise in the treatment of paroxysmal nocturnal hemoglobinuria (133). Furthermore, the factor B small molecule inhibitor iptacopan has recently demonstrated benefit for paroxysmal nocturnal hemoglobinuria (134) and IgA nephropathy (135). Therefore, should complement dysregulation be identified as a pathologic mechanism during long COVID, several available inhibitors could be tested in clinical trials. Despite the promise of complement therapeutics, it is important to note that modulation of complement may suppress the immune system and increase the risk for invasive bacterial and other infections (136–138). Therefore, additional investigation to understand the potential benefit of complement modulation during long COVID is necessary.

## Conclusion

Current evidence suggests that ongoing complement activation is a feature of long COVID and that complement-mediated tissue damage may contribute to its symptomatology. To date, these valuable findings remain sparse and heterogeneous, which probably reflects the diverse clinical presentation of long COVID, and more importantly, underscores the urgent need to examine the clinical validity of complement diagnostics and treatment. Several studies highlight the diagnostic potential of classical, alternative, and terminal pathway activation markers; however, many of these assays are not standardized and are only carried out at specialized laboratories, which complicates the comparison across each study and widespread clinical use. Future studies are needed to validate and refine a biomarker set that could be readily adopted for clinical testing. Longitudinal studies with consistent methodologies are necessary to define the dynamics of complement activation and understand the role of complement in disease development and progression. Moreover, while several studies have reported antibodies against herpesviruses and classical pathway activation as potential triggers, detailed mechanistic studies are necessary to establish a chain of events that lead to the observed ongoing changes in complement components in long COVID. These studies will enable the identification of patient subgroups more likely to benefit from complement therapy.

## Figures and Tables

**Table 1:**
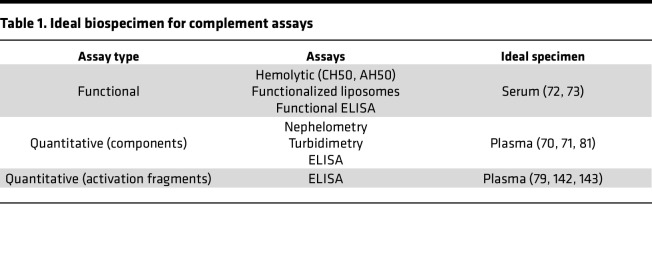
Ideal biospecimen for complement assays

**Table 2:**
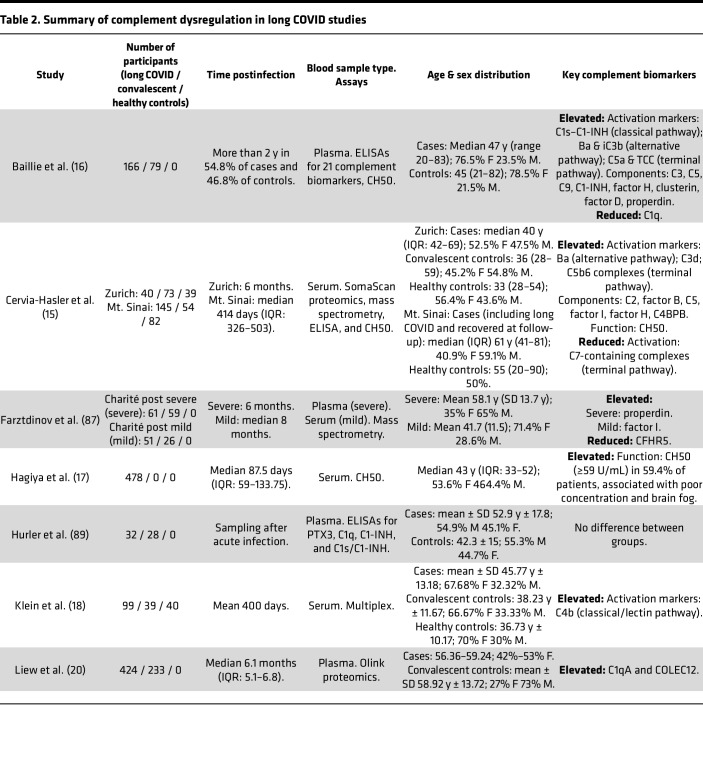
Summary of complement dysregulation in long COVID studies

## Version 1 08/22/2025

Electronic publication
